# Ajuba inhibits hepatocellular carcinoma cell growth via targeting of β-catenin and YAP signaling and is regulated by E3 ligase Hakai through neddylation

**DOI:** 10.1186/s13046-018-0806-3

**Published:** 2018-07-24

**Authors:** Min Liu, Ke Jiang, Guibin Lin, Peng Liu, Yumei Yan, Tian Ye, Gang Yao, Martin P. Barr, Dapeng Liang, Yang Wang, Peng Gong, Songshu Meng, Haozhe Piao

**Affiliations:** 1 Institute of Cancer Stem Cell, Dalian Medical University Cancer Center, 9 Lvshun Road South, Dalian, 116044 China; 2 Department of neurosurgery, Cancer Hospital of China Medical University, Liaoning Cancer Hospital & Institute, No. 44 Xiaoheyan Road, Dadong District, Shenyang, 110042 Liaoning Province China; 3 Huizhou No. 3 People’s Hospital, Affiliated Hospital of Guangzhou Medical University, No. 1 Xuebei Street, Qiaodong Road, Huizhou, 615000 China; 4 Department of Hepatobiliary Surgery, The First Affiliated Hospital, Dalian Medical University, No. 222 Zhongshan Road, Dalian, 116021 China; 5 The First Department of Ultrasound, The First Affiliated Hospital, Dalian Medical University, No. 222 Zhongshan Road, Dalian, 116021 China; 6 Thoracic Oncology Research Group, Institute of Molecular Medicine, Trinity Centre for Health Sciences, St. James’s Hospital & Trinity College, Dublin, Ireland; 7 Department of general surgery, Shenzhen University General Hospital, No. 1098 Xueyuan Road, Shenzhen, 518055 China; 8 Carson International Cancer Research Centre, Shenzhen University School of Medicine, No.3688 Nanhai Road, Shenzhen, 518060 China

**Keywords:** Ajuba, Hakai, β-Catenin, Neddylation, YAP, Hepatocellular carcinoma

## Abstract

**Background:**

Aberrant activation of β-catenin and Yes-associated protein (YAP) signaling pathways has been associated with hepatocellular carcinoma (HCC) progression. The LIM domain protein Ajuba regulates β-catenin and YAP signaling and is implicated in tumorigenesis. However, roles and mechanism of Ajuba expression in HCC cells remain unclear. The E3 ligase Hakai has been shown to interact with other Ajuba family members and whether Hakai interacts and regulates Ajuba is unknown.

**Methods:**

HCC cell lines stably depleted of Ajuba or Hakai were established using lentiviruses expressing shRNAs against Ajuba or Hakai. The effects of Ajuba on HCC cells were determined by a number of cell-based analyses including anchorage-independent growth, three dimension cultures and trans-well invasion assay. In vivo tumor growth was determined in a xenograft model and Ajuba expression in tumor sections was examined by immunohistochemistry. Co-immunoprecipitation, confocal microscopy and immunoblot assay were used to examine the expression and interaction between Ajuba and Hakai.

**Results:**

Depletion of Ajuba in HCC cells significantly enhanced anchorage-independent growth, invasion, the formation of spheroids and tumor growth in a xenograft model, suggesting a tumor suppressor function for Ajuba in HCC. Mechanistically, Ajuba depletion triggered E-cadherin loss and β-catenin translocation with increased Cyclin D1 levels. In addition, depletion of Ajuba upregulated the levels of YAP and its target gene CYR61. Furthermore, siRNA-mediated knockdown of either β-catenin or YAP attenuated the pro-tumor effects by Ajuba depletion on HCC cells. Notably, Ajuba stability in HCC cells was regulated by Hakai, an E3 ligase for E-cadherin. Hakai interacted with Ajuba via its HYB domain and induced Ajuba neddylation, which was antagonized by the neddylation inhibitor, MLN4924, but not MG132. We further show that overexpression of Hakai in HCC cells markedly increased anchorage-independent growth, spheroid-formation ability and tumor growth in xenografts whereas Hakai depletion resulted in these opposite effects, indicating an oncogenic role for Hakai in HCC. Hakai also induced β-catenin translocation with increased levels of Cyclin D1.

**Conclusions:**

Our data suggest a role for Ajuba and Hakai in HCC, and uncover the mechanism underlying the regulation of Ajuba stability.

**Electronic supplementary material:**

The online version of this article (10.1186/s13046-018-0806-3) contains supplementary material, which is available to authorized users.

## Background

Hepatocellular carcinoma (HCC) is one of the most common and lethal tumors worldwide. Despite the heterogeneous nature of human HCC, some altered signaling pathways involved in HCC have been discovered. Among them, Wnt/β-catenin cascade has emerged as a pivotal player in HCC [1, 2], detected in roughly 25% of the patients [3, 4]. β-catenin is the principal downstream effector of Wnt canonical activation. Although somatic mutations in the β-catenin and Axin1 genes are involved in the aberrant activation of the Wnt/β-catenin signaling in HCC [1, 5], cytoplasmic accumulation and/or nuclear translocation of β-catenin were found to be independent of β-catenin mutations in the subclass of more aggressive tumors [4]*.* In addition to β-catenin signaling, the emerging role of the Hippo tumor suppressor cascade in liver tumorgenesis has been well established [6, 7]. YAP and transcriptional co-activator with PDZ-binding motif (TAZ), two transcriptional co-activators, are the main downstream effectors of the mamalian Hippo signaling pathway. Upon activation, the hippo core kinase cascade phosphorylates YAP/TAZ, leading to their cytoplasmic localization and proteolysis [6]. A growing number of studies document the oncogenic roles of YAP as well as TAZ in liver tumorgenesis and progression [8–13].

Ajuba belongs to the Ajuba family which contains three members with overlapping tissue/cell expression: Ajuba, LIM domain containing protein 1 (LIMD1), and Wilms tumor 1 interacting protein (WTIP) [14, 15]. The Ajuba family of proteins is characterized by the presence of a unique N terminal region, the pre-LIM region, and three tandem C-terminal LIM domains [15]. Previous reports showed that Ajuba negatively regulates the Wnt signaling pathway by promoting GSK-3β-mediated phosphorylation of β-catenin [16]. In addition, Ajuba is required for Rac activation and maintenance of E-cadherin adhesion [17]. In epithelial cells, Ajuba is recruited to newly forming adherens junctions through an interaction with α-catenin, thereby stabilizing junctions [15]. Therefore, Ajuba is involved in a diverse array of cellular processes such as cell-to-cell adhesion, cell migration, cell proliferation and mitosis/cytokinesis [15]. Of note, accumulating evidence demonstrating frequent inactivating mutations in Ajuba in cutaneous squamous cell carcinoma and esophageal squamous cell carcinoma [18–20], and loss-of-function alterations of Ajuba in head and neck squamous cell carcinomas [21] suggests that Ajuba may be involved in tumorigenesis. Indeed, it has been shown that Ajuba functions as a potential tumor suppressor in small cell lung cancer and in malignant mesothelioma [22, 23]. Conversely, Ajuba plays an oncogenic role in cutaneous squamous cell carcinoma and in colorectal cancer [18, 24], suggesting a cell type-specific role of Ajuba in cancer cells. In hepatocellular carcinoma, however, the role of Ajuba remains largely unknown.

Ajuba functions in cancer through targeting of diverse signaling pathways. For instance, Ajuba promotes colorectal cancer cell survival through suppression of JAK1/STAT1 signaling [25]. In esophageal squamous cell carcinoma cells, Ajuba upregulates MMP10 and MMP13 expression to promote migration and invasion [26]. In addition, mounting evidence indicate that the Hippo pathway is highly involved in Ajuba activity in cancer. Given that Ajuba family of LIM proteins have been identified as negative regulators of the Hippo pathway [27], Ajuba is shown to positively regulate YAP oncogenic activity in several cancers [28, 29]. However, there is also evidence demonstrating that Ajuba suppresses YAP activity to inhibit malignant mesothelioma cell proliferation [23], suggesting that similar to the role of Ajuba in cancer, Ajuba-regulated YAP activity may be cancer cell specific.

Hakai is a Casitas B-lineage lymphoma (Cbl)-like ubiquitin ligase that mediates ubiquitination of E-cadherin upon Src activation and regulates E-cadherin complex endocytosis [30–32]. Hakai-mediated down-regulation of E-cadherin is involved in oncogenic and/or tumor-suppressive signaling pathways such as RACK1 and Slit-Robo signaling during tumor progression [31, 33, 34]. In addition to targeting E-cadherin, Hakai reportedly promotes breast cancer cell proliferation in an E-cadherin independent manner [35], and elevated in human colon and gastric adenocarcinomas [35–37]. A recent study reported that Hakai is involved in CD147-mediated HCC progress via E-cadherin ubiquitination and degradation [38]. However, the direct role of Hakai in HCC has not been defined.

In this study, we investigated the role of Ajuba, in addition to Hakai, in HCC cells. We demonstrate that Ajuba functions as a tumor suppressor in HCC cells in vitro and in a xenograft model, while Hakai acts as an oncoprotein. Notably, we show that Ajuba stability is regulated by Hakai in HCC cells via neddylation.

## Methods

### Cell lines and transfection

The cell lines, 293 T, COS7, Hep3B, HepG2, Huh7, SK-Hep1, SMMC7721 and SNU449 were obtained from the American Type Cell Culture (ATCC, Manassas, VA) and cultured according to ATCC guidelines. MHCC97H, MHCC97L and HCCLM3 cell lines were cultured in DMEM (Gibco), BEL7402 cells were cultured in RPMI-1640 (Gibco). All medium were supplemented with 10% FBS and cultured in a humidified incubator under 5% CO_2_ at 37 °C. Transfection of plasmids into all cells was performed using Lipofectamine 2000 (Invitrogen), according to the manufacturer’s instructions.

### Plasmids and adenoviruses

Flag-tagged Hakai was kindly provided by Prof. Y Fujita [30]. Myc-tagged Hakai, GFP-tagged Hakai and deletion mutants (ΔPro, ΔRing, ΔpTyr-B, ΔHYB, Pro and Ring) were producted by PCR. Myc-tagged Ajuba and deletion mutants (Lim and Prelim) were used as previously described [39]. GFP-tagged β-Catenin was purchased from Addgene (NO. 28009). The AdEasy XL adenoviral vector system (Stratagene) was used to generate the adenovirus, adenoviruses expressing GFP-tagged Hakai. The control virus (AdVector) was constructed as previously described [40].

### Antibodies and reagents

Anti-Flag, GFP, Ajuba (for immunohistochemistry), GAPDH and Cycloheximide were purchased from Sigma, while anti-Cyclin D1, anti-CYR61 and anti-Hakai were purchased from Santa Cruz. The following antibodies were used; anti-Ajuba (for immunoblotting, Cell Signaling Technology), anti-E-Cadherin and anti-β-Catenin (Abcam), anti-Myc-tagged antibody (Invitrogen), anti-YAP (NOVUS, NB110–58308) proteasome inhibitor MG132 and neddylation inhibitor MLN4924 (Selleckchem) and the proteasome inhibitor Lactacystin (LAC) was obtained from R&D Sytems. Drugs were dissolved in 0.5% dimethyl sulfoxide (DMSO) as stock solutions and stored at − 20 °C.

### Bioinformatics analysis

Normalized gene expression data for the entire 115 Singapore HCC cohort was obtained from Gene Expression Omnibus (GEO) data repository with accession number GSE76427 [41]; Statistical analyses were performed using the R statistical software version 3.2.2. Spearman’s correlation between Ajuba and YAP mRNA expression was calculated. Survival analysis was conducted via the ‘survival’ R package. HCC patients are categorized into High and Low Ajuba expression group using the 1rd quartile as cutoff points (1th quartile vs. quartiles 2–4) and survival curves were based on Kaplan-Meier estimates. When multiple probes were mapped to the same gene, probe with most significant correlation and log-rank test *p* value was selected to represent the gene.

### RNA interference

RNA interference was used to knock down GSK3β, β-Catenin and YAP. Two siRNA oligonucleotides of GSK3β were used: 5’-CUCAAGAACUGUCAAGUAATT-3′; 5’-GGAAUAUGCCAUCG GAUATT-3′. Two siRNA oligonucleotides of β-Catenin were used: 5’-GGAUGUGGAUACCUCCCAATT-3′; 5’-CCAUUACAACUCUCC ACAATT-3′. Two siRNA oligonucleotides of Ajuba were used: 5’-GGACCGGGAUUAUCACUUUTT-3′; 5’-CCAAGUAUACUGUGUCACCTT-3′. A scrambled siRNA: 5’-UUCUCCGAACGUGUCACGU TT-3′ was used as a negative control. The silencing efficiency was detected by immunoblot assay.

### Lentiviral constructs and stable cell lines

The lentiviral constructs, Hakai (CBLL1) shRNA (sc-89,853-V), Ajuba shRNA (sc-60,066-V) and non-coding shRNA (sc-108,080) were purchased from Santa Cruz. Lentiviral particles were used to directly infect HepG2 and BEL7402 cells and stable clones were selected using puromycin (3 μg/mL for BEL7402 and 1.5 μg/mL for HepG2). Stable expression of Myc-tagged Ajuba and Flag-tagged Hakai cell lines were established as previously described [42].

### Colony formation and 3D cultures

To determine colony formation, cells were cultured in complete medium for 14 days. Colonies were counted by light microscopy. For 3D cultures, cells were seeded (600 cells/well) in ultra-low attachment 96-well plates and maintained in serum-free DMEM/F12 medium supplemented with 20 ng/ml epidermal growth factor (EGF), 10 ng/ml basic fibroblast growth factor (bFGF) and B27 (B27 and medium were mixed in a volume ratio of 1:50). Seven days after seeding, propagated spheroid bodies were imaged and counted by light microscopy [43].

### Trans-well invasion assay

Cell suspension (200 μL) was seeded at 1 × 10^5^ cells/well to the upper chamber, while media containing 10% FBS (650 μL) was added to the lower chamber. After 24 h, the non-penetrated cells were removed using a cotton swab. Cells that had invaded the back of the membrane of the trans-well chamber were stained with 0.1% crystal violet after fixed by 4% formaldehyde. The invasive capacity of cells was defined according to the total number of cells in randomly selected fields by light microscopy.

### Immunoprecipitation and immunoblotting

Immunoprecipitation (IP) and immunoblotting (IB) were performed as previously described [40]. To quantify changes in protein expression, the densitometries of protein bands were determined using a calibrated GS-670 densitometer. For endogenous interactions, cells grown in 10 cm^2^ dishes were harvested and the cell lysates were then subjected to IP.

### Confocal microscopy

For confocal microscopy, cells were seeded on coverslips (NEST, 801008) for 24 h. Cells were fixed in 4% paraformaldehyde (PFA), permeabilized in 0.2% Triton X-100 and incubated in 3% Bovine Serum Albumin (BSA). Cells were then incubated with primary antibody for 2 h at room temperature followed by incubation with secondary antibody for 0.5 h at room temperature. Nuclei were stained with 5 μg/mL DAPI (Sigma) in PBS. Images were acquired using a confocal microscope (Leica TCS SP5×) using a ×60 oil objective. Images from each experiment were acquired using the same exposure time during the same imaging session [44].

### Immunohistochemistry

Immunohistochemistry was performed on 4 mm thick paraffin-embedded tissue sections. Briefly, processed sections of Ajuba protein were incubated with the anti-Ajuba antibody (1:200), The DAB Detection Kit was used to develop staining signal according to the protocols provided for the streptavidin-perosidase system (Sangon Biotech, China). The slides were counterstained with haematoxylin. All sections were investigated by light microscopy. Neoplastic cells that both exhibited cytoplasmic immunoreactivity with clearly brown-yellow were regarded as Ajuba positive staining.

### In vivo xenograft model

HCC cells (1 × 10^7^) were injected subcutaneously into flanks of nude mice (5–6 weeks old). Tumor growth was monitored using calipers where two perpendicular tumor diameters were measured weekly and the tumor volume calculated using the formula: volume = (greatest diameter) × (smallest diameter)^2^/2. After 5 weeks, all experimental mice were sacrificed with ether anesthesia. Tumor were harvested and imaged. Animal experiments were conducted at Dalian Medical University (Dalian, China), in compliance with the national guidelines for the care and use of laboratory animals. All animal experiments were approved by the experimental animal ethics committee of Dalian Medical University.

### Statistical analysis

Comparisons of data were first performed using one-way analysis of variance (ANOVA). Multiple comparisons between treatment groups and controls were evaluated using Dunnett’s least significant difference (LSD) test. For analysis of in vivo data, statistical significance between groups was calculated based on the LSD test using SPSS 17.0 software (SPSS Inc., Chicago, IL, USA). A *p*-value of *p* < 0.05 was considered statistically significant. All experiments were carried out in triplicate as three independent experiments.

## Results

### Ajuba depletion enhances HCC cells growth in vitro and in vivo

**Fig. 1:**
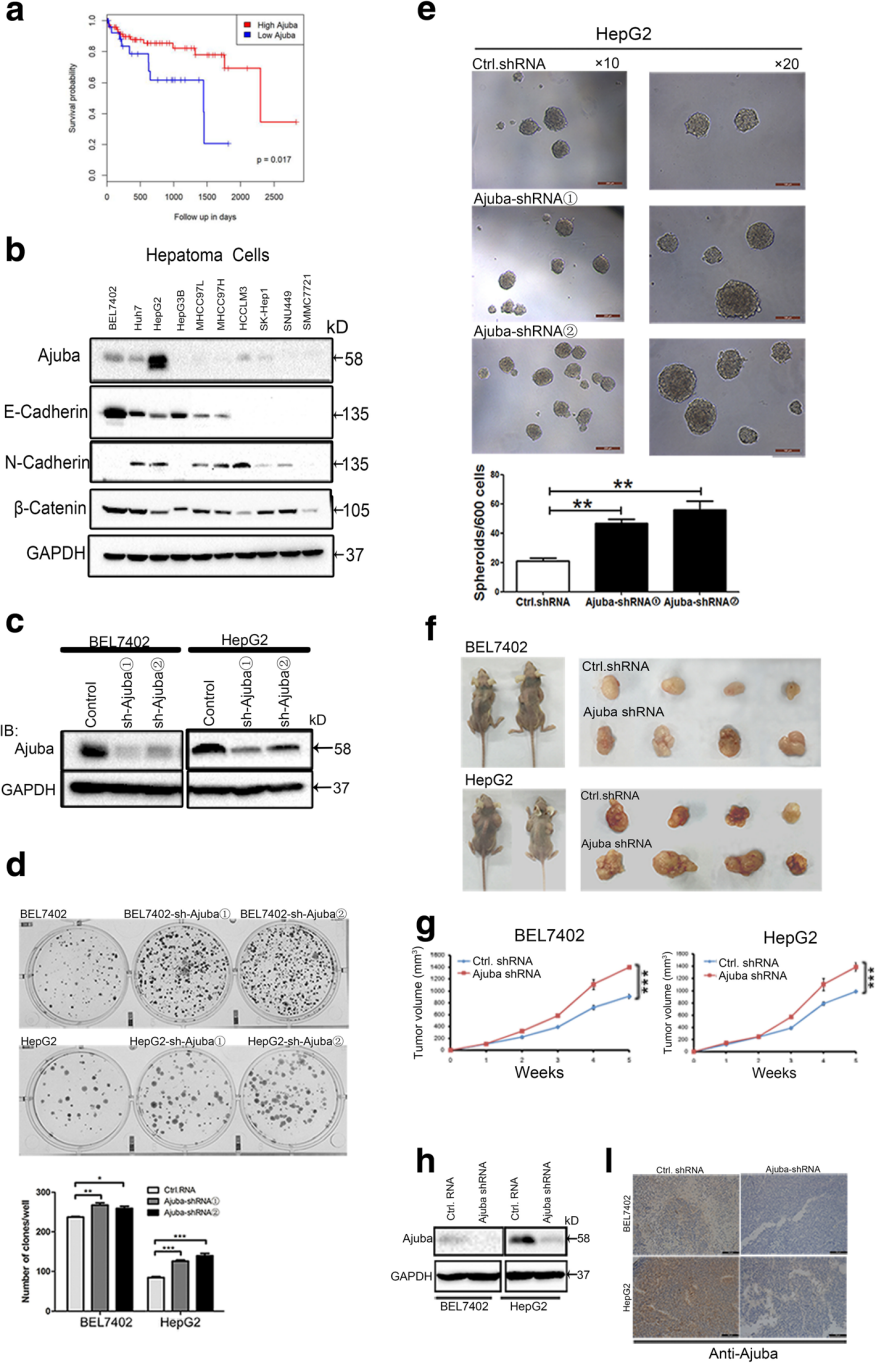
Depletion of Ajuba potentiates hepatocellular carcinoma cell growth in vitro and in vivo. **a** Negative correlation of mRNA expression of AJUBA and YAP in HCC Patient. **b** Immunoblot (IB) analysis of Ajuba and E-cadherin-associated protein levels in hepatocellular carcinoma (HCC) cell lines. GAPDH was used as a loading control. **c** Immunoblot analysis of the efficiency of stable knockdown of Ajuba in BEL7402 and HepG2 cells using GAPDH as a loading control. IB, immunoblot. **d**, **e** Analysis of the ability of Ajuba-depleted BEL7402 and HepG2 cells to form colonies (**d**) and spheroids in three dimensional (3D) cultures (**e**). Scale bar = 100 μm (×20), 200 μm (×10). **f** Grossly visible tumor size in Ajuba-depleted HCC cells vs control mice, measured on the day of sacrifice (after 5 weeks). **g** Enlarged tumor volumes in Ajuba-depleted HCC cells vs control mice, measured weekly. **h**, **i** Immunoblot assay (**h**) or immunohistochemistry assay (**i**) for expressions of Ajuba proteins in Ajuba shRNA or negative shRNA groups of xenograft model. Scale bar = 50 μm. Data are presented as Mean ± SEM from three independent experiments (*p < 0.05, **p < 0.01, ***p < 0.001)

To investigate whether Ajuba is clinically relevant to HCC, we assessed how Ajuba affects clinical outcomes using a microarray data (GEO accession: GSE76427) [41]. Kaplan–Meier survival analysis demonstrated that low level of Ajuba was a strong indicator for an inferior overall survival (OS) (*P* values was 0.017) in a 115 HCC patient cohort (Fig. 1a), suggesting a significantly unfavorable prognosis and shorter life span. We next examined the function of Ajuba in HCC in cell culture models. Endogenous protein levels of Ajuba and several E-cadherin-associated proteins were measured in a number of HCC cell lines by immunoblot analysis. Ajuba protein was largely undetectable in the majority of cell lines examined (Fig. 1b), except for HepG2 cells, and to a lesser extent, BEL7402 and Huh7 cells, indicating that Ajuba is expressed at low levels in HCC cells. The two cell lines BEL7402 and HepG2 were then selected for stable depletion of endogenous Ajuba by lentivrus-mediated shRNA. Control cells were infected with a lentivrus carrying a scrambled shRNA (Fig. 1c). Functionally, colony formation assays showed that depletion of Ajuba in HCC cells markedly enhanced anchorage-independent growth compared to control cells (Fig. 1d). Moreover, Ajuba-depleted HepG2 cells showed significantly enhanced spheroid-forming ability compared to control cells when cultured in three dimensional (3D) conditions (Fig. 1e). To assess the physiological relevance of our in vitro findings, we further extended our investigation in a xenograft model. Nude mice inoculated with Ajuba-depleted BEL7402 or HepG2 cells showed a significant increase in tumor growth compared to control groups (Fig. 1f and g). Depletion of Ajuba in tumor sections was examined by immunoblot assay and immunohistochemistry respectively (Fig. 1h and j). Collectively, these data demonstrated that depletion of Ajuba increases the growth of HCC cells both in vitro and in in vivo*,* suggesting a tumor suppressor function of Ajuba in HCC.

### β-Catenin translocation and activity as well as YAP signaling are induced in HCC cell lines in response to Ajuba depletion

**Fig. 2:**
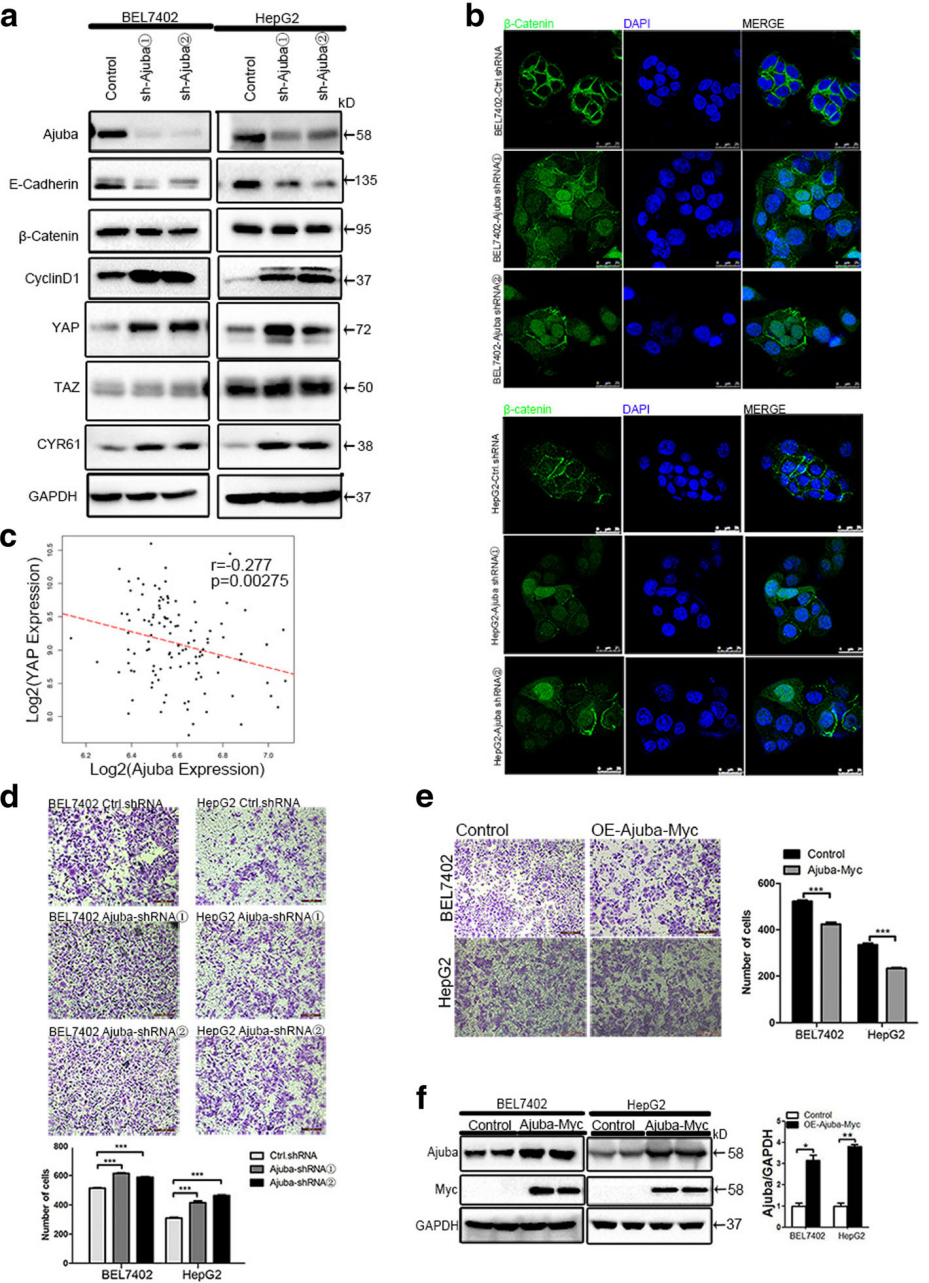
Ajuba depletion induces β-catenin translocation and Cyclin D1 expression in HCC cell lines. **a** Immunoblotting with specific antibodies against Ajuba, E-cadherin, β-catenin, Cyclin D1, YAP, TAZ and CYR61 in Ajuba-depleted BEL7402 and HepG2 cells. GAPDH was used as a loading control. The ratios of expression E-Cadherin to their corresponding GAPDH are represented. **b** Ajuba-depleted HCC cells were fixed for immunofluorescence and stained for β-catenin protein (green) and DAPI (blue). Representative merged images are also shown for fluorescence signals. Scale bar = 25 μm. **c** Correlation of Ajuba expression with OS in HCC. Low expression of Ajuba was associated with worse OS compared to high expression of Ajuba. Kaplan-Meier curves and log-rank test were used to evaluate OS. *P* < 0.05 was considered significant. **d**, **e** Representative images and quantification of migration and invasion of Ajuba-depleted (**d**) or Ajuba-overexpressing (**e**) HCC cells. Scale bar = 200 μm. HCC, hepatocellular carcinoma. **f** Expression in response to the overexpression of constructs of Ajuba-Myc was examined by IB, the ratios of expression Ajuba to their corresponding GAPDH are represented. Data are presented as Mean ± SEM from three independent experiments (*p < 0.05, **p < 0.01, ***p < 0.001)

Previous studies indicated that Ajuba is required for maintenance of E-cadherin adhesion [17]. In addition, Ajuba associates with β-catenin and negatively regulates the Wnt signaling pathway in HeLa cells [16]. Given the well-recognized role of β-catenin in HCC, we hypothesized that Ajuba may regulate β-catenin activity in HCC cells. Immunoblot analysis demonstrated that E-cadherin protein levels were significantly down-regulated in Ajuba-depleted BEL7402 and HepG2 cells, while β-catenin levels remained unchanged (Fig. 2a). However, Immunofluorescence staining demonstrated that in Ajuba-depleted HCC cells, β-catenin decreased from cell-to-cell contacts, increasing in both the cytoplasm and nucleus (Fig. 2b), while control cells showed the typical honeycomb effect of cell surface β-catenin staining (Fig. 2b). Furthermore, Cyclin D1 expression levels were also increased in both Ajuba-depleted HCC cells (Fig. 2a). This is in agreement with Cyclin D1 as being a well-established WNT/β-catenin targeted gene. Our data thus suggest that Ajuba negatively regulates β-catenin-mediated transcription, consistent with previous findings in HeLa cells [16]. In addition to the induction of β-catenin translocation, depletion of Ajuba resulted in a robustly increase in YAP levels as detected by immunoblot assay while TAZ levels were not altered (Fig. 2a). CYR61 is a well-known YAP-targeted gene [6]. We noticed a strong elevation of CYR61 levels in Ajuba-depleted HCC cells (Fig. 2a). Interestingly, Spearman correlation analysis on the same microarray data (GEO accession: GSE76427) in Fig. 1a showed a significant negative correlation between Ajuba and YAP mRNA level (*p* = 0.00275; *r* = − 0.277) (Fig. 2c). Thus, Ajuba depletion induces enhanced β-catenin and YAP signaling in the tested HCC cells.

Depletion of Ajuba-mediated loss of E-cadherin in HCC cells suggests that Ajuba may have an effect on cell invasion. Stable depletion of Ajuba migrate and to invade Matrigel (Fig. 2d). Conversely, ectopic expression of Ajuba in either BEL7402 or HepG2 cell lines significantly decreased the number of the cells invading and migrating through the Matrigel-coated membrane of the chamber compared to vector-transfected cells (Fig. 2e). Expression of Myc-Ajuba in BEL7402 and HepG2 cell lines were confirmed by immunoblot assay (Fig. 2f).

It is known that GSK3β regulates β-catenin activity in classical WNT/β-catenin pathway. To investigate whether Ajuba negatively regulates β-catenin activity via GSK3β, we transfected specific siRNAs to silence GSK3β protein in Ajuba-depleted HCC cell lines. The knockdown of GSK3β was confirmed by immunoblot assay (Additional file 1: Figure S1A). We observed that knockdown of GSK3β did not affect β-catenin translocation to the nucleus (Additional file 1: Figure S1B) in Ajuba-depleted HCC cell lines compared to control siRNA (Additional file 1: Figure S1B). Importantly, depletion of Ajuba-induced CyclinD1 expression was not affected by GSK3β knockdown (Additional file 1: Figure S1A). Together, these data suggests that Ajuba may directly regulate beta-Catenin signaling in HCC cells.

We further investigated whether altered β-catenin activity is responsible for the function of Ajuba in HCC cells. As shown in Additional file 1: Figure S1C, depletion of Ajuba-induced CyclinD1 expression in HCC cell lines was diminished by knockdown of β-catenin with siRNAs targeting β-catenin. Moreover, β-catenin knockdown significantly decreased the colony formation in Ajuba-depleted HCC cell lines compared with control siRNAs (Additional file 1: Figure S1D). Interestingly, the colony formation in Ajuba-depleted HCC cell lines was substantially inhibited when YAP was knockdown (Additional file 1: Figure S1E).

### Ajuba interacts with Hakai

**Fig. 3:**
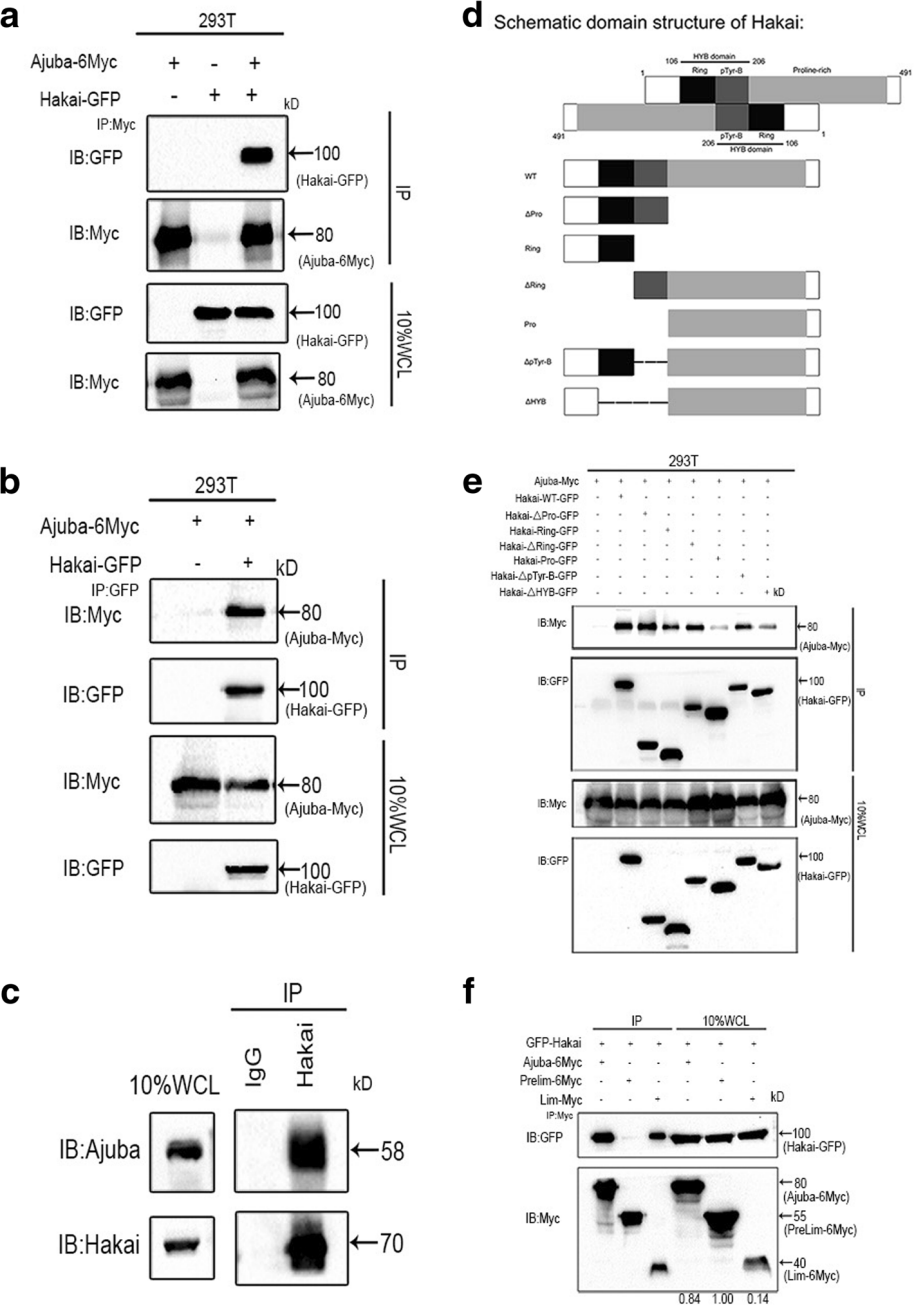
Hakai interacts with Ajuba through its HYB domain. **a**, **b** 293 T cells transfected with Myc-tagged Ajuba alone, GFP-tagged Hakai alone or both in combination. Cell lysates were subjected to immunoprecipitation (IP) with anti-Myc-tag (**a**) or anti-GFP-tag antibody (**b**) and immnoblotted (IB) with the indicated antibodies. **c** Detection of interaction between endogenous Hakai and Ajuba by IB at in BEL7402 cells (**d**, **e**). Schematic showing Hakai deletion mutants and their relative abilities to interact with Ajuba in transfected 293 T cells. **f** Co-IP of 293 T cells showing the domain of Ajuba binding to Hakai. IB, immnoblot. IP, immunoprecipitation. WCL, Whole-cell lysates

Our data suggest a tumor suppressor role of Ajuba in HCC cells, we are interested in how Ajuba level is regulated in HCC cells. The E3-ubiquitin ligase Hakai, mediates ubiquitination of E-cadherin and is found to be complexed with LIMD1, a member of Ajuba family [32]. In addition, WTAP, another Ajuba family member, was shown to interact with Hakai in previous studies [45, 46]. We thus hypothesized that Hakai may interact with Ajuba and regulate Ajuba turnover. We first examined the interaction of the two proteins by IP in HEK 293 T cells. Exogenously expressed Ajuba tagged with the Myc epitope (Myc-Ajuba) and GFP-tagged Hakai (GFP-Hakai), were reciprocally co-immunoprecipitated (Fig. 3a and b). Furthermore, the physiologic association between endogenous Ajuba and Hakai was detected in BEL7402 cells (Fig. 3c).

Hakai contains three domains: a RING finger, a short pTyr-B domain and a proline-rich domain [30]. Recently Sivaraman and colleagues identified a new HYB (Hakai pTyr-binding) domain in Hakai, where this domain consists of a Ring domain and the pTyr-B domain [32] (Fig. 3d). To map the regions of interaction between Ajuba and Hakai, we generated a series of GFP-tagged Hakai deletion constructs. Deletion of either the Ring domain or the pTyr-B domain in Hakai did not markedly affect its binding to Ajuba (Fig. 3e). However, deletion of the HYB domain dramatically diminished its interaction with Ajuba, indicating that the HYB domain in Hakai is responsible for its association with Ajuba. For Ajuba, pre-LIM domain-only did not interact with GFP-Hakai, while LIM domain-only was sufficient for its interaction with Hakai (Fig. 3f). Confocal microscopy analysis revealed the co-localization between ectopic Ajuba and Hakai in the cytoplasm of HepG2 cells (Additional file 2: Figure S2A). Interestingly, GFP-Hakai localized in both the cytoplasm and nucleus when coexpressed with the vector control (Additional file 2: Figure S2A), similar to observations by other studies [15, 17]. Thus, our data suggest that Ajuba overexpression might induce the alteration of Hakai localization. Collectively, these data indicates that Hakai does indeed interact with Ajuba.

### Hakai promotes Ajuba degradation

**Fig. 4:**
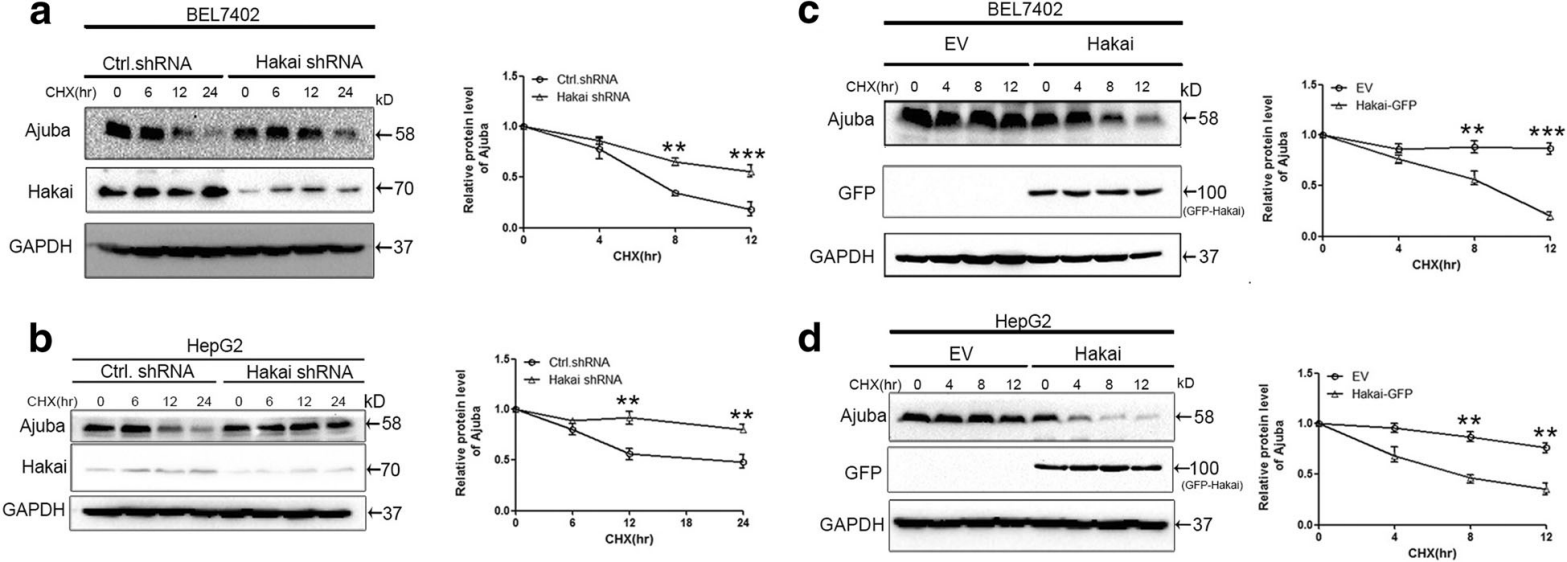
Hakai regulates Ajuba protein stability in HCC cells. **a**, **b** Immublotanalysis of Ajuba protein levels in Hakai-depleted or control HCC cells (A: BEL7402; B: HepG2) in the presence of cycloheximide (CHX, 80 μg/ml) for the indicated times, right panels showing the quantification of Ajuba relative protein levels. GAPDH was used as a loading control. **c**, **d** HCC cells were infected with controls or Hakai adenovirus and treated with CHX for the indicated times. Ajuba protein levels were determined by immunoblotting and quantified. (**c**: BEL7402; **d**: HepG2). GAPDH was used as a loading control. Data are presented as Mean ± SEM from three independent experiments (**p < 0.01, ***p < 0.001)

We next examined whether Hakai regulates Ajuba turnover in HCC cells. To test our hypothesis, we examined endogenous Ajuba protein levels in the absence or presence of Hakai. We established BEL7402 and HepG2 cells that were stably depleted of Hakai using lentivrus-mediated shRNA. The half-life of Ajuba protein was monitored in the presence of cycloheximide (CHX), which blocks de novo protein synthesis. Depletion of Hakai markedly prolonged the half-life of Ajuba in BEL7402 (Fig. 4a) and HepG2 (Fig. 4b) cells. Quantification of Ajuba protein levels was determined and analyzed statistically (Fig. 4a and b, right panels). Conversely, adenovirus-mediated expression of GFP-tagged Hakai considerably shortened the half-life of Ajuba in both cell lines compared to vector controls (Fig. 4c and d, right panels showing the statistic analysis of the quantification of Ajuba protein levels). The half-life of ectopic Ajuba in HEK293T cells in the presence of CHX was consistently reduced by Hakai over-expression (Additional file 3: Figure S3A). Together, these data imply that Hakai promotes Ajuba degradation.

### Hakai regulates Ajuba stability via neddylation

**Fig. 5:**
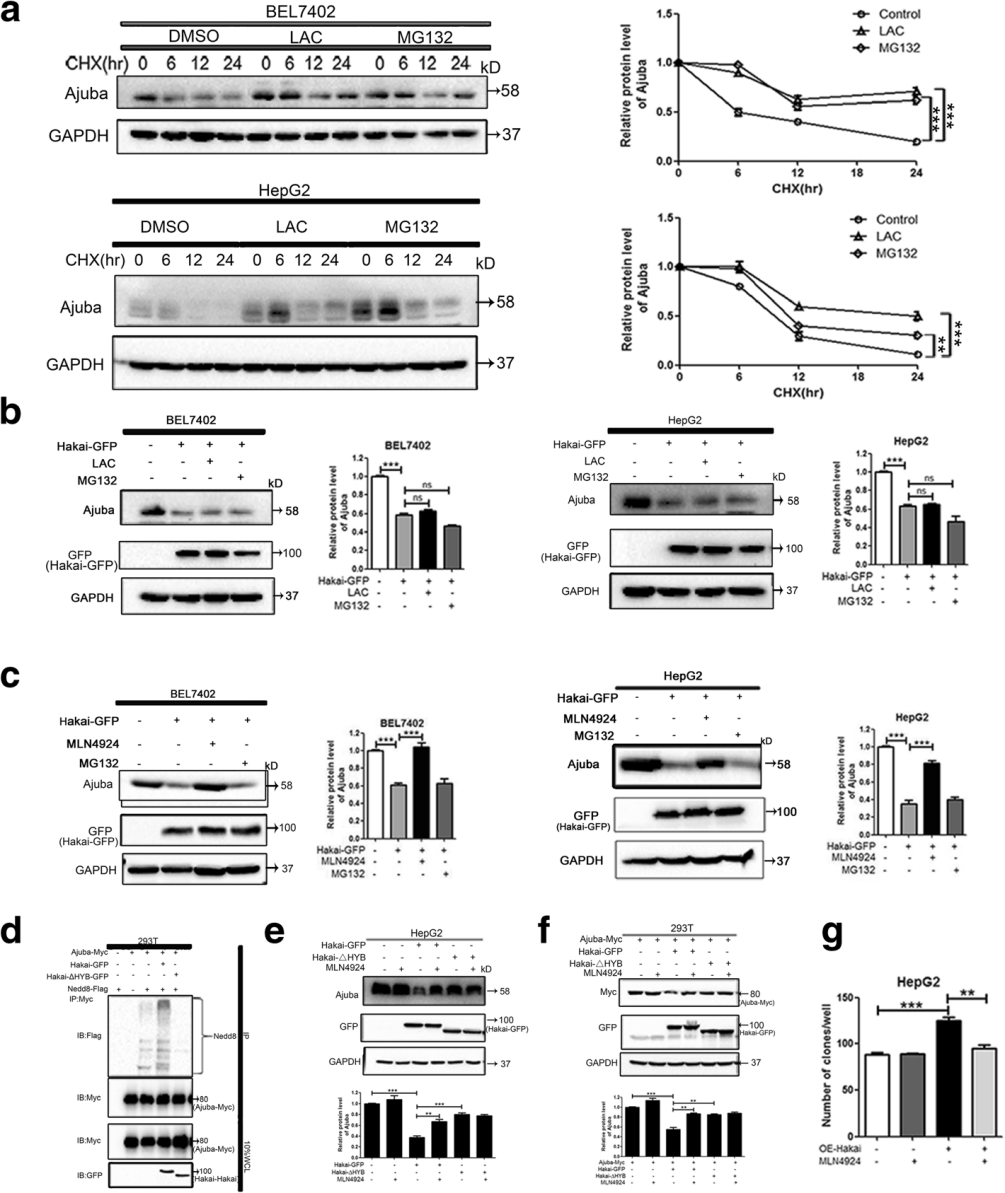
Hakai mediates Ajuba degradation via neddylation. **a** Immunoblot analysis and quantification of the half-life of Ajuba in the presence of cycloheximide (CHX, 80 μg/ml), and in the presence or absence of lactacystin (LAC, 20 μM) or MG132 (10 μM) in BEL7402 and HepG2 cells. GAPDH was used as a loading control. Data are presented as Mean ± SEM from three independent experiments (**p < 0.01, ***p < 0.001). **b**, **c** BEL7402 or HepG2 cells were transfected with GFP-tagged Hakai and treated with 0.5% DMSO, 20 μM LAC, 10 μM MG132 (**b**) or 5 μM MLN4924 (**c**). Endogenous Ajuba levels were determined by immunoblotting using anti-Ajuba antibody. GAPDH was used as a loading control. Densitometry was performed for quantification, and the ratios of Ajuba and GAPDH are presented. **d** Neddylation assay of Ajuba in 293 T cells transfected with the indicated plasmids. IB, immnoblot. IP, immunoprecipitation. WCL, Whole-cell lysates. **e** HepG2 cells were transfected with GFP-tagged Hakai or HYB domain deletion mutant and treated with 5 μM MLN4924 or 0.5% DMSO. Endogenous Ajuba protein levels were determined and quantified. GAPDH was used as a loading control. **f** Ectopic Ajuba protein levels were examined in 293 T cells transfected with Myc-tagged Ajuba, GFP-tagged Hakai or HYB domain deletion mutant in the presence or absence of 5 μM MLN4924. GAPDH was used as a loading control. **g** Colony formation assay performed in HepG2 cells infected with Adenoviruses expressing Hakai in presence or absence of 5 μM MLN4924. Data are presented as Mean ± SEM from three independent experiments (**p < 0.01, ***p < 0.001)

To elucidate the potential mechanism of Ajuba degradation by Hakai, we first investigated whether Hakai mediates Ajuba protein stability. Treatment with the proteasome inhibitors lactacystin (LAC) or MG132, substantially extended the half-life of endogenous Ajuba in BEL7402 and HepG2 cells (Fig. 5a, right panels showing statistical analyses of the quantification of Ajuba protein level), suggesting that the degradation of Ajuba in HCC cells is proteasome-dependent. As expected, ectopic expression of Hakai triggered degradation of endogenous Ajuba in HCC cells (Fig. 5b). However, neither LAC nor MG132 blocked ectopic Hakai-induced Ajuba degradation (Fig. 5b, right panels showing statistical analyses of the quantification of Ajuba protein level), indicating that Hakai-mediated Ajuba degradation may not occur via the classical ubiquitination pathway. Intriguingly, treatment with MLN4924, an investigational inhibitor of Nedd8 activating enzyme (NAE) that inhibits the activity of all Cullin E3 ligases [47, 48], markedly decreased the effect of ectopic Hakai on Ajuba degradation in BEL7402 and HepG2 cells (Fig. 5c, right panels showing statistical analyses of the quantification of Ajuba protein level). This finding points towards the involvement of the neddylation pathway. Likewise, MLN4924 prolonged the half-life of endogenous Ajuba in BEL7402 and HepG2 cells in the presence of CHX (Additional file 4: Figure S4A, right panels showing statistical analyses of the quantification of Ajuba protein level). To examine whether Hakai could affect the ubiquitination of Ajuba, 293 T cells were co-transfected with the indicated plasmids in Additional file 4: Figure S4B. We observed that over-expression of Hakai did not markedly affect the ubiquitination of Myc-Ajuba (Additional file 4: Figure S4B, lane 4 vs lane 3).

To investigate whether Hakai neddylates Ajuba, an in vivo neddylation assay was performed in 293 T cells in which protein expression levels of both Ajuba and Hakai were not detectable by immunoblot analysis (data not shown). Basal neddylation of Myc-Ajuba was detected even in the absence of ectopic Hakai (Additional file 4: Figure S4C, lane 3). However, over-expression of Hakai considerably enhanced neddylation of Myc-Ajuba (Additional file 4: Figure S4C, lane 4 vs lane 3). Furthermore, deletion of the HYB domain in Hakai markedly decreased the neddylation levels of Ajuba compared to cells transfected with wild type (WT) Hakai (Fig. 5d, compare lane 4 with 5), indicating that the HYB domain of Hakai is required for Hakai-mediated Ajuba neddylation. Moreover, compared with WT Hakai, the HYB domain deletion mutant significantly blunted the degradation of endogenous Ajuba in HepG 2 cells and ectopic Ajuba in 293 T cells (Fig. 5e, lane 3 vs lane 5; Fig. 5f, lane 3 vs lane 5; lower panels showing statistical analyses of the quantification of Ajuba protein levels), indicating that in addition to mediating neddylation, the HYB domain of Hakai is critical for Hakai-mediated Ajuba degradation.

To evaluate the biological consequences of Hakai-mediated Ajuba degradation, we examined whether Hakai regulates Ajuba activity in HCC cells. Adenovirus-mediated ectopic expression of Hakai in HepG2 cells led to significantly increased colony formation, which was markedly blocked by pre-treatment with MLN4924 (Fig. 5g). As our data indicated that Ajuba deletion induced β-catenin translocation into nucleas in HCC cells, we examined whether this effect is Hakai depedent. As illustrated in Additional file 5: Figure S5A, knockdown of Ajuba by two different siRNAs resulted in evident translocation of β-catenin into nucleas in Hakai-depleted HepG2 cells. The knockdown effiency was assayed by immunoblotting (Additional file 5: Figure S5B). Interestingly we observed a reverse expression pattern of Ajuba and Hakai in BEL7402 as well as in HepG2 cells (Additional file 5: Figure S5C).

### Hakai interacts with β-catenin and induces its translocation

**Fig. 6:**
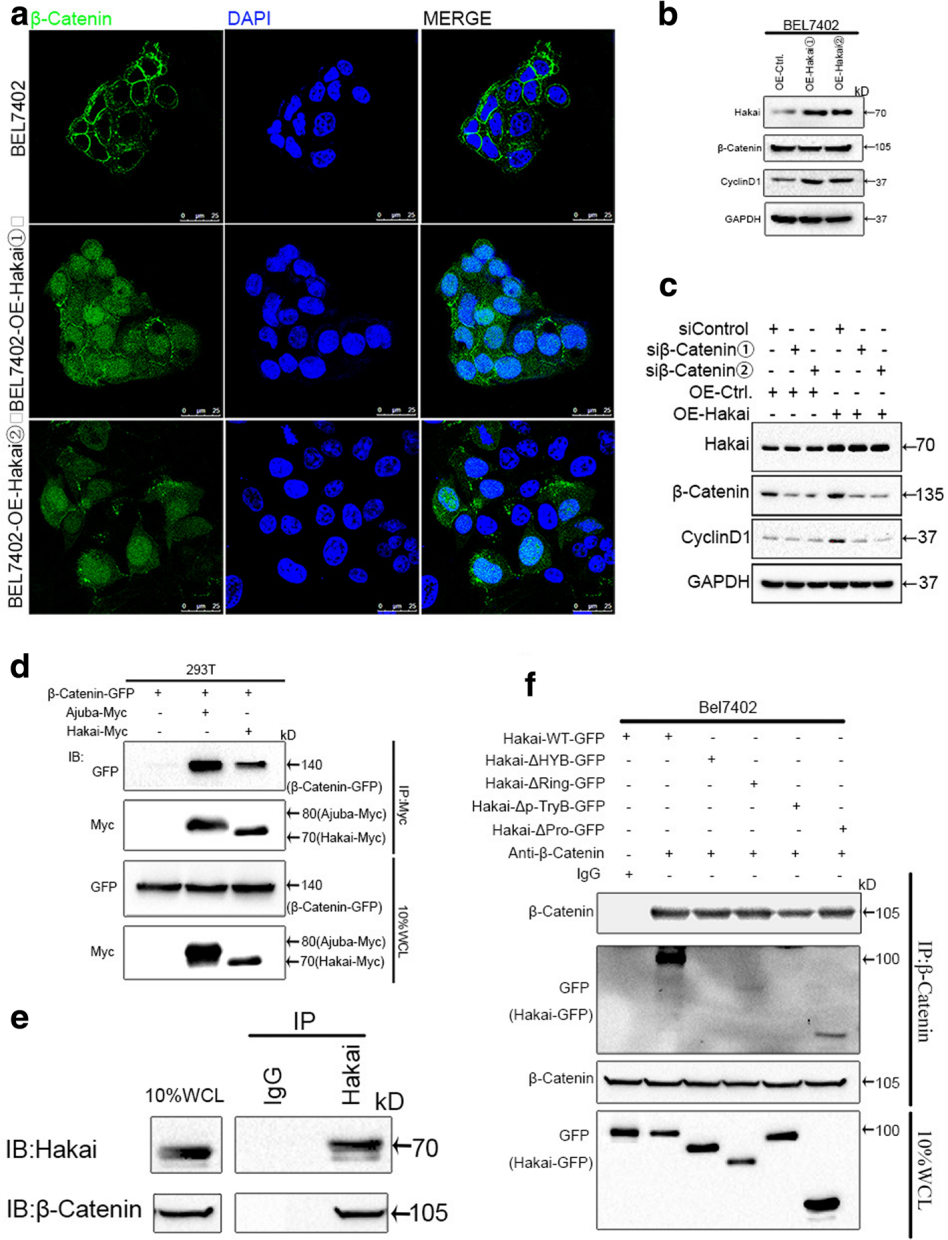
Hakai associates with β-catenin and induces its translocation. **a** BEL7402 cells stably expressing Flag-tagged Hakai were fixed for immunofluorescence and stained for β-catenin protein (green) and DAPI (blue). Representative merged images showing overlap of fluorescence signals are shown. Scale bar = 25 μm. **b** Immunoblot analysis of β-catenin, Cyclin D1 and Hakai in BEL7402 cells stably expressing Hakai. GAPDH was used as a loading control. The ratios of expression Hakai and Cyclin D1 to their corresponding GAPDH are represented. Data are presented as Mean ± SEM from three independent experiments (***p < 0.001). **c** BEL7402 cells of stably expressing Hakai or vector control were transfected with two siRNA duplexex targeted to β-Catenin (siβ-Catenin) or control siRNA (siControl) for 48 h. Cell lysates were analyzed by immunoblotting for Hakai, β-catenin and Cyclin D1 protein expression. GAPDH was used as a loading control. **d** Co-immunoprecipitaion (IP) of GFP-tagged β-catenin and Myc-tagged Hakai or Myc-tagged Ajuba in 293 T cells. **e** IP of endogenous Hakai and Ajuba in BEL7402 cells with anti-Hakai antibody. **f** Co-IP of wild-type Hakai (WT) or its deletion mutants and endogenous β-catenin in transfected BEL7402 cells. IB, immunoblot. IP, immunoprecipitation. WCL, Whole-cell lysates. The results shown are representative of three separate experiments

We observed β-catenin translocation to cytoplasm and nucleus in BEL7402 cells stably expressing Flag-tagged Hakai (Fig. 6a). Notably, Cyclin D1 protein levels were elevated in Hakai-overexpressing BEL7402 cells (Fig. 6b), indicating that Hakai positively regulates β-catenin activity in HCC cells. Similar results were found in SNU449 cells in which Ajuba protein was not detectable (data not shown). Furthermore, knockdown of β-catenin with siRNAs diminished the expression of CyclinD1 in BEL7402 cells stably expressing Hakai compared with control siRNAs (Fig. 6c).

To dissect the underlying mechanism by which Hakai regulates β-catenin activity, the interaction of Hakai with β-catenin was examined. To this end, 293 T cells were transfected with GFP-tagged β-catenin alone, or in combination with either Myc-tagged Hakai or Myc-tagged Ajuba. Co-immunoprecipitation assays indicated that Myc-Hakai was associated with GFP-β-catenin (Fig. 6d). Consistent with previous reports [16], Myc-Ajuba was also shown to interact with β-catenin (Fig. 6d). Endogenous Hakai was also able to interact with endogenous β-catenin in BEL7402 cells (Fig. 6e). Domain mapping analysis showed that Hakai interacts with endogenous β-catenin via the HYB domain (Fig. 6f). Taken together, these data confirm the interaction of Hakai with β-catenin.

### Hakai promotes HCC growth in vitro and in vivo

**Fig. 7:**
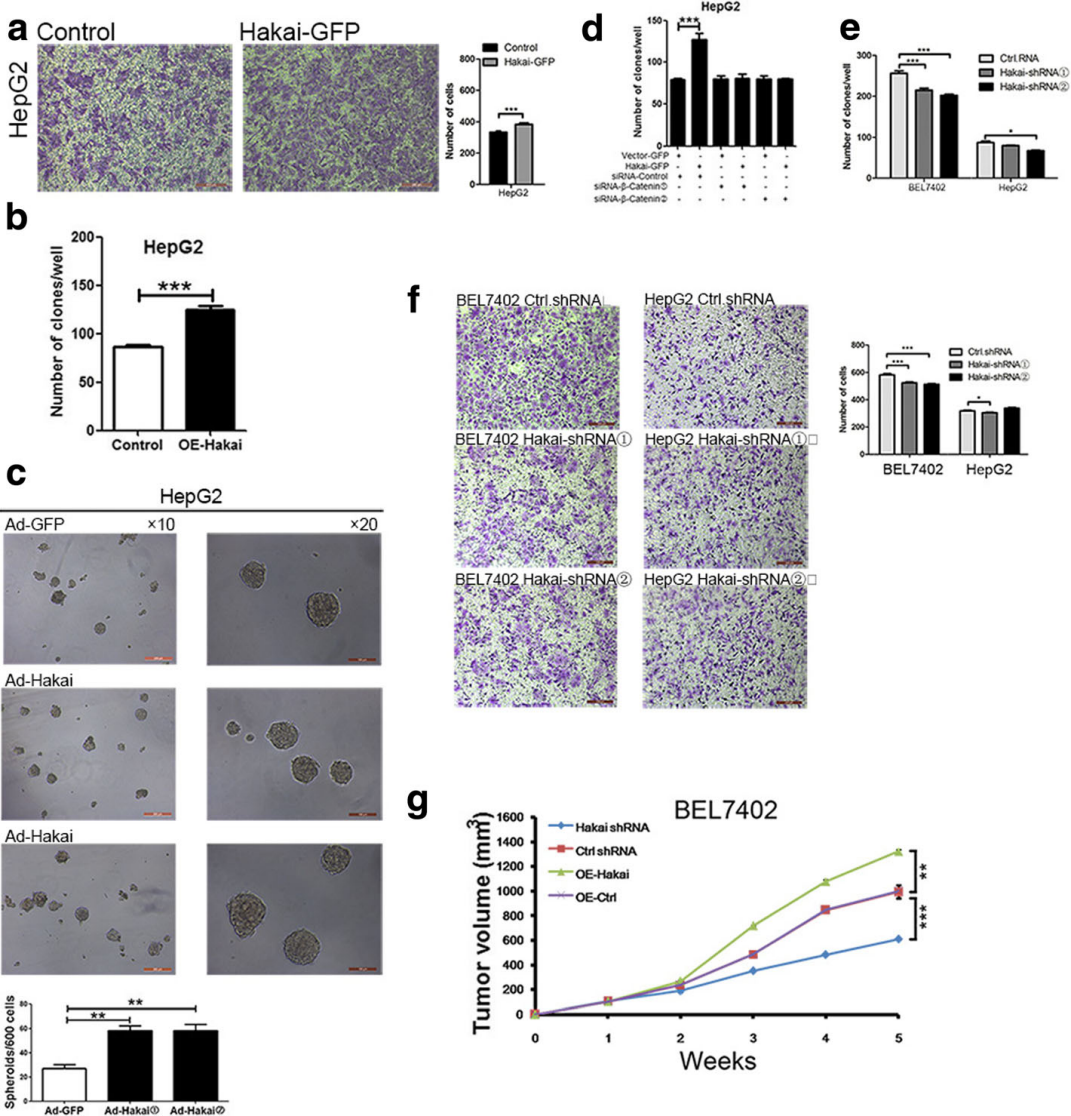
Hakai promotes HCC growth in vitro and in vivo. **a** Representative images and quantification of invasion in GFP-tagged Hakai-overexpressing HepG2 cells by adenovirus. Scale bar = 200 μm. **b**, **c** Analysis of the ability of Hakai-overexpressing HepG2 cells by adenovirus to form colonies (**b**) and spheroids in three dimensional conditions (**c**). Scale bar = 100 μm (×20), 200 μm (×10). **d** HepG2 cells were overexpressed with GFP-Vector or GFP-Hakai by adenovirus for 24 h, and then cell were transfected with two siRNA duplexex targeted to β-Catenin (siβ-Catenin) or control siRNA (siControl). Cells were cultured in complete medium for 12 days during colony formation assays. **e** Quantification of colony forming ability of Hakai-depleted BEL7402 and HepG2 cells, or control cells. **f** Representative images and quantification of cell invasion of Hakai-depleted BEL7402 and HepG2 cells. Scale bar = 200 μm. **g** Tumor volumes in mice inoculated with Hakai-depleted and Hakai-overexpressing cells vs control mice, as measured weekly. Data are presented as Mean ± SEM from three independent experiments (**p < 0.01, ***p < 0.001)

Whether Hakai plays a role in the growth of HCC was subsequently examined. Hakai-mediated effects on HCC cell growth and invasion was initially examined. In doing so, adenovirus-mediated expression of GFP-tagged Hakai in HepG2 cells significantly increased the ability of these cells to invade in matrigel assays, and to form colonies and spheroids in 3D cultures compared with control cells (Fig. 7a, b and c). However, the effect of Hakai in HepG2 cells was blocked by knowndown of β-catenin with siRNAs (Fig. 7d). Similar results were obtained in BEL7402 cells infected with recombinant adenoviruses expressing Hakai (Additional file 6: Figure S6A and S6B). On the contrary, BEL7402 and HepG2 cells stably depleted of Hakai displayed a significant decrease in their ability to invade and form colonies relative to that seen in control cells (Fig. 7e and f).

To confirm the above in vitro findings, xenograft tumor growth assays were carried out using BEL7402 stable cell lines expressing or depleted of Hakai. Hakai-expressing cells inoculated in nude mice showed a significant increase in tumor growth compared with control cells (Fig. 7g), whereas tumors derived from Hakai-depleted BEL7402 cells were significantly smaller in volume compared to those formed from corresponding control cells (Fig. 7g).

## Discussion

In this study, we have shown that depletion of Ajuba triggers loss of E-cadherin, nuclear translocation of β-catenin and increased YAP expression in HCC cells, thus promoting HCC cell growth in vitro and in a xerograft model. These findings would suggest that Ajuba functions as a tumor suppressor in HCC. We further show that Ajuba protein turnover in HCC cells is mediated by E3 ubiquitin ligase Hakai via neddylation, while Hakai promoted HCC cell growth both in vitro and in vivo. To our knowledge, this is the first report revealing the role of Ajuba as well as Hakai in HCC.

While the role of Ajuba in cancer has been documented [18, 22–24], this remains controversial. Recent studies demonstrate that Ajuba is up-regulated in colon cancer cell lines and acts as a tumor-promoting gene in colon cancer [24, 49]. In addition, Ajuba has been documented to play an oncogenic role in pancreatic cancer cells [49] and esophageal squamous cell carcinoma cells [26]. In contrast to the oncogenic role of Ajuba in certain types of cancer, Ajuba has been shown to suppress cell proliferation of MM cells [23]. Others have shown that Ajuba inhibits SCLC cell growth, while its repression strongly correlates with shorter survival in SCLC patients [22]. Our current data show that Ajuba functions as a tumor suppressor in HCC cells. Therefore, these studies, together with our current data, suggest a cell-type specific role for Ajuba in cancer cells. We further explored the underlying mechanisms mediated by Ajuba in HCC cells and showed that depletion of Ajuba in HCC cells triggers loss of E-cadherin and translocation of β-catenin, in addition to increased Cyclin D1 levels. This is in line with previous studies showing that Ajuba negatively regulates the Wnt/β-catenin signaling pathway in HeLa cells [16]. In addition, other study has shown that the Hippo/YAP pathway plays a role in Ajuba-mediated anti-tumor effects in MM [23]. In our study, substantial elevation of YAP together with its target gene, CYR61, was detected in Ajuba-depleted HCC cells. Furthermore, YAP knockdown diminished the pro-tumor effects of Ajuba depletion on HCC cells. Therefore, our data suggest that Hippo pathways are involved in Ajuba-mediated anti-tumor effects in HCC cells, consistent with the reported oncogenic role of YAP in HCC [9, 10, 50–53].

One of the most interesting findings in the present study is that Hakai mediates Ajuba turnover via neddylation. The control of Ajuba stability is largely unknown and no E3 ligase has been identified that specifically targets Ajuba. We found that MLN4924, an inhibitor of neddylation, but not MG132 or LAC, attenuated Hakai-induced degradation of Ajuba in HCC cells, suggesting that neddylation rather than ubiquitination is involved in Hakai-mediated Ajuba turnover. Hakai structurally relates to the E3 ubiquitin ligase c-Cbl. Of interest, previous studies reported that c-Cbl mediates the neddylation of epidermal growth factor receptor (EGFR) and transforming growth factor β (TGF-β) type II receptor [54, 55]. Thus, similar to c-Cbl, Hakai may regulate target protein via neddylation in addition to ubiquitination. Interestingly, although it is generally accepted that Hakai mediated E-cadherin ubiquitination for proteasomal degradation [10–12], a recent study demonstrated that CD147 overexpression in HCC cells enhances E-cadherin ubiquitination by recruiting Hakai for lysosomal degradation, which is prevented by chloroquine (an inhibitor of lysosomal degradation) treatment, but not MG132 [38]. Therefore, these studies including ours highlight the complex pattern for Hakai-mediated protein degradation.

**Fig. 8:**
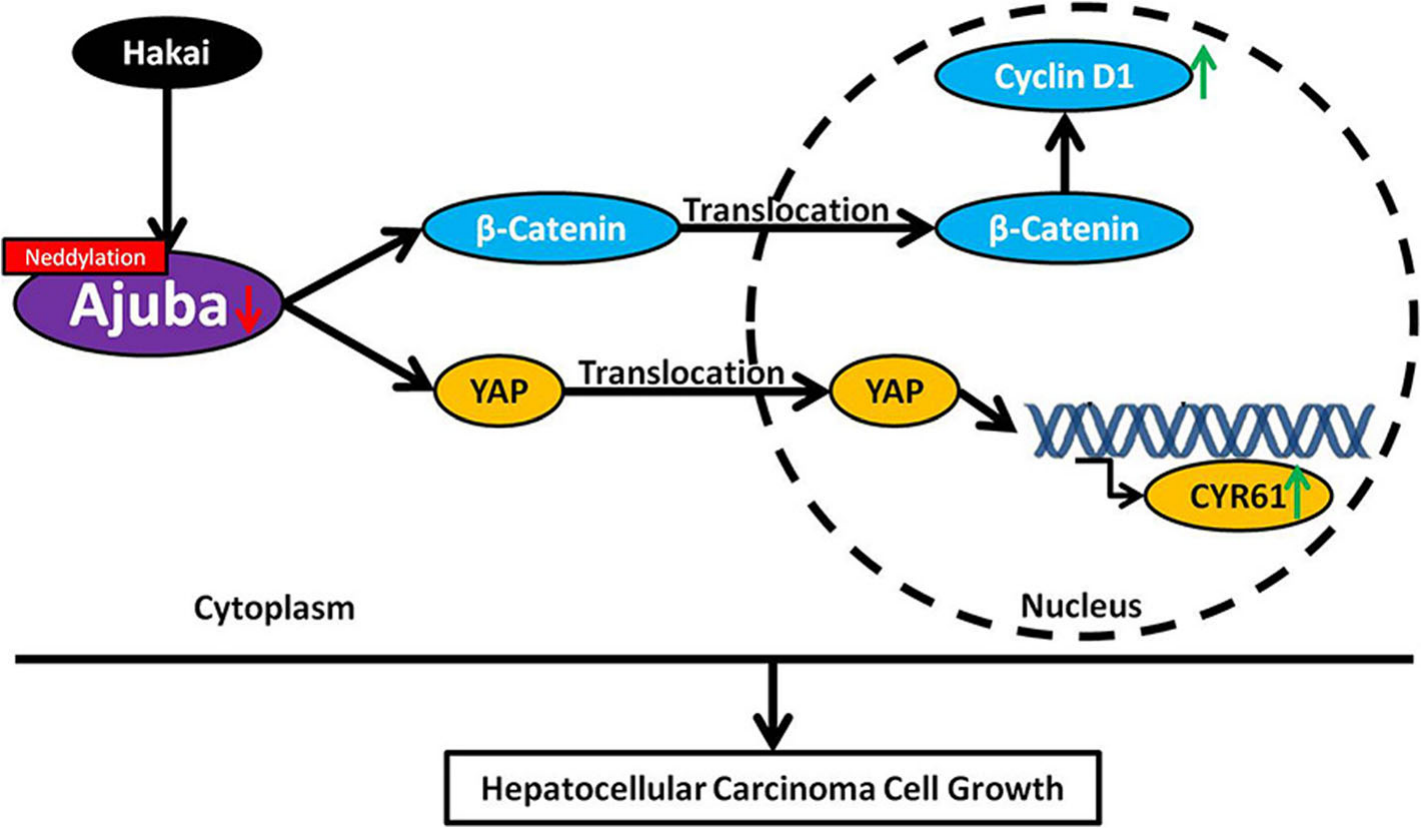
A model depicting the proposed mechanism for Ajuba-mediated effects on HCC cell growth and its regulation by Hakai. A working model illustrating that Ajuba-knockdown induced β-catenin translocation and activity as well as YAP signaling to promote HCC cells growth. In addition, Hakai promoted Ajuba degradation via neddylation

We further explored the underlying mechacnism of Hakai-mediated Ajuba neddylation and degradation. Mechanistically, Hakai interacts with Ajuba with its HYB domain and induces Ajuba neddylation. Therefore, our data highlight a potential mechanism by which Ajuba stability is regulated. We thus propose a schematic working model depicting the mechanism for Ajuba-mediated effects on HCC cell growth and its regulation by Hakai (Fig. 8). It shoulded be noted that knockdown of Ajuba-induced translocation of β-catenin into nucleas in HCC cells is not Hakai-dependent.

Hakai has been implicated in cancers such as colon adenocarcinomas [34, 37, 56], although its role in breast cancer is controversial [35, 57]. Although a recent study reported Hakai-mediated E-cadherin ubiquitination and lysosomal degradation in the role of Hakai in HCC has not, as yet, been documented. Collectively, our data show for the first time in HCC that Hakai promotes the growth of HCC cells and tumors, while mechanistically, Hakai interacts with β-catenin and induces its nuclear translocation, thereby identifying an oncogenic role for Hakai in the development of HCC.

## Conclusions

Taken together, we found that knockdown of Ajuba promoting HCC cell proliferation in vitro and in a xerograft model. Our results prove a novel mechanism that Ajuba regulates the protein level of E-cadherin, nuclear translocation of β-catenin and increased YAP expression in HCC cells. We further show that Ajuba protein turnover in HCC cells is mediated by E3 ubiquitin ligase Hakai via neddylation, while Hakai enhanced HCC cell proliferation both in vitro and in vivo. This study may suggest a novel strategy for HCC treatment.

## Additional files

Additional file 1: **Figure S1.** The regulation of β-Catenin and cell growth in HCC cells. (A, B) HCC cells were transfected with specific siRNAs to silence GSK3β protein in Ajuba-depleted HCC cell lines. The expression of GSK3β, Ajuba, CyclinD1 and GAPDH were tested by immunoblot assay (A). β-Catenin translocation were tested by confocal assay, Scale bar = 25 μm (B). (C, D) HCC cells were transfected with specific siRNAs to silence β-Catenin protein in Ajuba-depleted HCC cell lines. The expression of β-Catenin, Ajuba, CyclinD1 and GAPDH were tested by immunoblot assay (C). Cell growth was tested by colony formation (D). (E) HCC cells were transfected with specific siRNAs to silence YAP protein in Ajuba-depleted HCC cell lines. Cell growth was tested by colony formation. Data are presented as Mean ± SEM from three independent experiments (***p < 0.001). (JPG 515 kb)

Additional file 2: **Figure S2.** Ajuba was co-localized with Hakai in HepG2 cells. (A) HepG2 cells were co-transfected with Myc-Ajuba or Myc-Vector and GFP-Hakai for 24 h. Cells were analyzed for GFP-Hakai/Myc-Ajuba co-localization, Scale bar = 25 μm. (JPG 97 kb)

Additional file 3: **Figure S3.** The half-life of ectopic Ajuba in HEK293T cells by Hakai over-expression. (A) HEK293T cells were infected with controls or Hakai adenovirus and treated with CHX for the indicated times. Ajuba protein levels were determined by immunoblotting and quantified. GAPDH was used as a loading control. Data are presented as Mean ± SEM from three independent experiments (*p < 0.05). (JPG 76 kb)

Additional file 4: **Figure S4.** Hakai mediates Ajuba degradation via neddylation. (A) Immunoblot analysis and quantification of the half-life of Ajuba in the presence of cycloheximide (CHX, 80 μg/ml), and in the presence or absence of MLN4924 (5 μM) in BEL7402 and HepG2 cells. GAPDH was used as a loading control. (B) Ubiquitination (Ub) assay of Ajuba in 293 T cells transfected with the indicated plasmids. (C) Neddylation assay of Ajuba in 293 T cells transfected with the indicated plasmids. IB, immnoblot. IP, immunoprecipitation. WCL, Whole-cell lysates. (JPG 103 kb)

Additional file 5: **Figure S5.** Ajuba knockdown-mediated β-catenin translocation into nucleus is not dependent on Hakai**.** (A, B) HepG2 cells were transfected with specific siRNAs to silence Ajuba protein in Hakai-depleted HepG2 cells. β-catenin translocation were tested by confocal assay, Scale bar = 25 μm (A). The expression of Ajuba, Hakai and β-catenin were tested by immunoblot assay, GAPDH was used as a loading control (B). (C) Immunoblot analysis of Ajuba and Hakai in BEL7402 and HepG2 cell lysis. GAPDH was used as a loading control. (JPG 617 kb)

Additional file 6: **Figure S6.** Hakai promotes BEL7402 cells invasion and growth. (A) Representative images and quantification of invasion in GFP-tagged Hakai-overexpressing BEL7402 cells by adenovirus. Scale bar = 200 μm. (B) Analysis of the ability of Hakai-overexpressing BEL7402 cells by adenovirus to form colonies. Data are presented as Mean ± SEM from three independent experiments (**p < 0.01, ***p < 0.001). (JPG 146 kb)

## Abbreviations

3D: Three dimensional
BSA: Bovine Serum Albumin
Cbl: Casitas B-lineage lymphoma
CHX: Cycloheximide
EGFR: Epidermal growth factor receptor
HCC: Hepatocellular carcinoma
IB: Immunoblotting
IP: Immunoprecipitation
LAC: lactacystin
LIMD1: LIM domain containing protein 1
MM: Malignant mesothelioma
NAE: Nedd8 activating enzyme
PFA: Paraformaldehyde
SCLC: Small cell lung cancer
TAZ: Transcriptional co-activator with PDZ-binding motif
TGF-β: Transforming growth factor β
WT: Wild type
WTIP: Wilms tumor 1 interacting protein
YAP: Yes-associated protein
