# Supplementary figures and images for: Ajuba inhibits hepatocellular carcinoma cell growth via targeting of β-catenin and YAP signaling and is regulated by E3 ligase Hakai through neddylation
Source: J Exp Clin Cancer Res. 2018 Jul 24;37:165. doi: 10.1186/s13046-018-0806-3 (PMC6057013; doi:10.1186/s13046-018-0806-3)

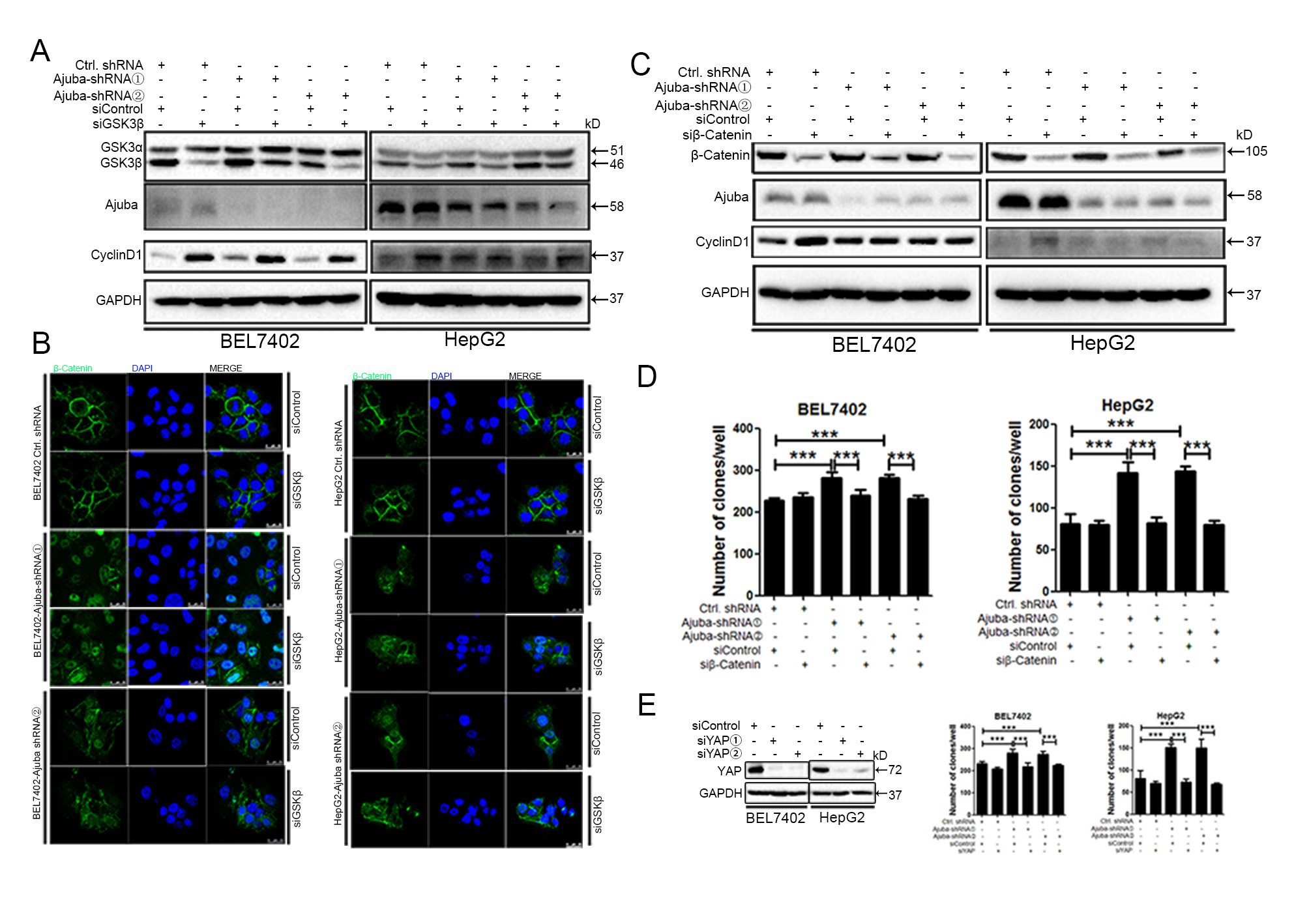

Supplement: Supplementary file 1 — Figure S1. The regulation of β-Catenin and cell growth in HCC cells. (A, B) HCC cells were transfected with specific siRNAs to silence GSK3β protein in Ajuba-depleted HCC cell lines. The expression of GSK3β, Ajuba, CyclinD1 and GAPDH were tested by immunoblot assay (A). β-Catenin translocation were tested by confocal assay, Scale bar = 25 μm (B). (C, D) HCC cells were transfected with specific siRNAs to silence β-Catenin protein in Ajuba-depleted HCC cell lines. The expression of β-Catenin, Ajuba, CyclinD1 and GAPDH were tested by immunoblot assay (C). Cell growth was tested by colony formation (D). (E) HCC cells were transfected with specific siRNAs to silence YAP protein in Ajuba-depleted HCC cell lines. Cell growth was tested by colony formation. Data are presented as Mean ± SEM from three independent experiments (***p < 0.001). (JPG 515 kb) [file 13046_2018_806_MOESM1_ESM.jpg]

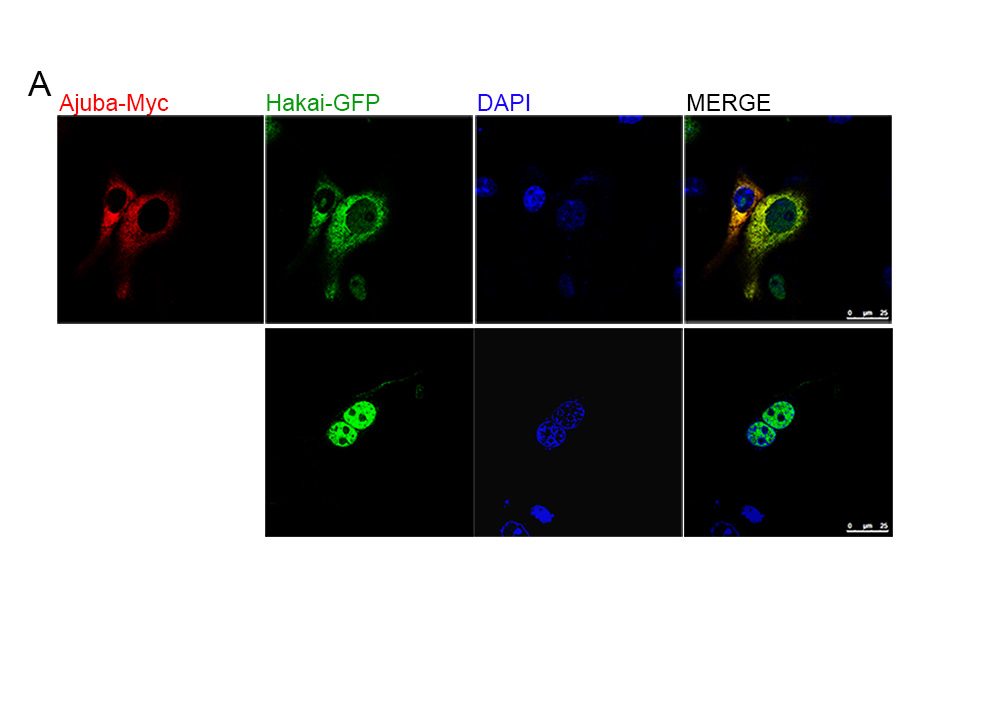

Supplement: Supplementary file 2 — Figure S2. Ajuba was co-localized with Hakai in HepG2 cells. (A) HepG2 cells were co-transfected with Myc-Ajuba or Myc-Vector and GFP-Hakai for 24 h. Cells were analyzed for GFP-Hakai/Myc-Ajuba co-localization, Scale bar = 25 μm. (JPG 97 kb) [file 13046_2018_806_MOESM2_ESM.jpg]

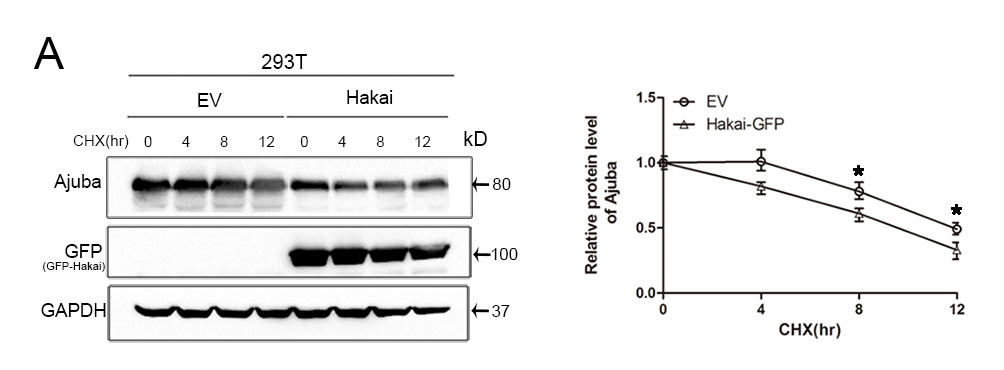

Supplement: Supplementary file 3 — Figure S3. The half-life of ectopic Ajuba in HEK293T cells by Hakai over-expression. (A) HEK293T cells were infected with controls or Hakai adenovirus and treated with CHX for the indicated times. Ajuba protein levels were determined by immunoblotting and quantified. GAPDH was used as a loading control. Data are presented as Mean ± SEM from three independent experiments (*p < 0.05). (JPG 76 kb) [file 13046_2018_806_MOESM3_ESM.jpg]

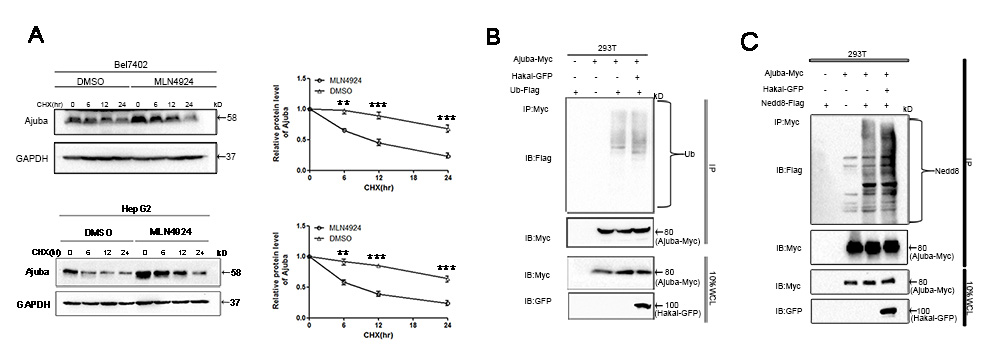

Supplement: Supplementary file 4 — Figure S4. Hakai mediates Ajuba degradation via neddylation. (A) Immunoblot analysis and quantification of the half-life of Ajuba in the presence of cycloheximide (CHX, 80 μg/ml), and in the presence or absence of MLN4924 (5 μM) in BEL7402 and HepG2 cells. GAPDH was used as a loading control. (B) Ubiquitination (Ub) assay of Ajuba in 293 T cells transfected with the indicated plasmids. (C) Neddylation assay of Ajuba in 293 T cells transfected with the indicated plasmids. IB, immnoblot. IP, immunoprecipitation. WCL, Whole-cell lysates. (JPG 103 kb) [file 13046_2018_806_MOESM4_ESM.jpg]

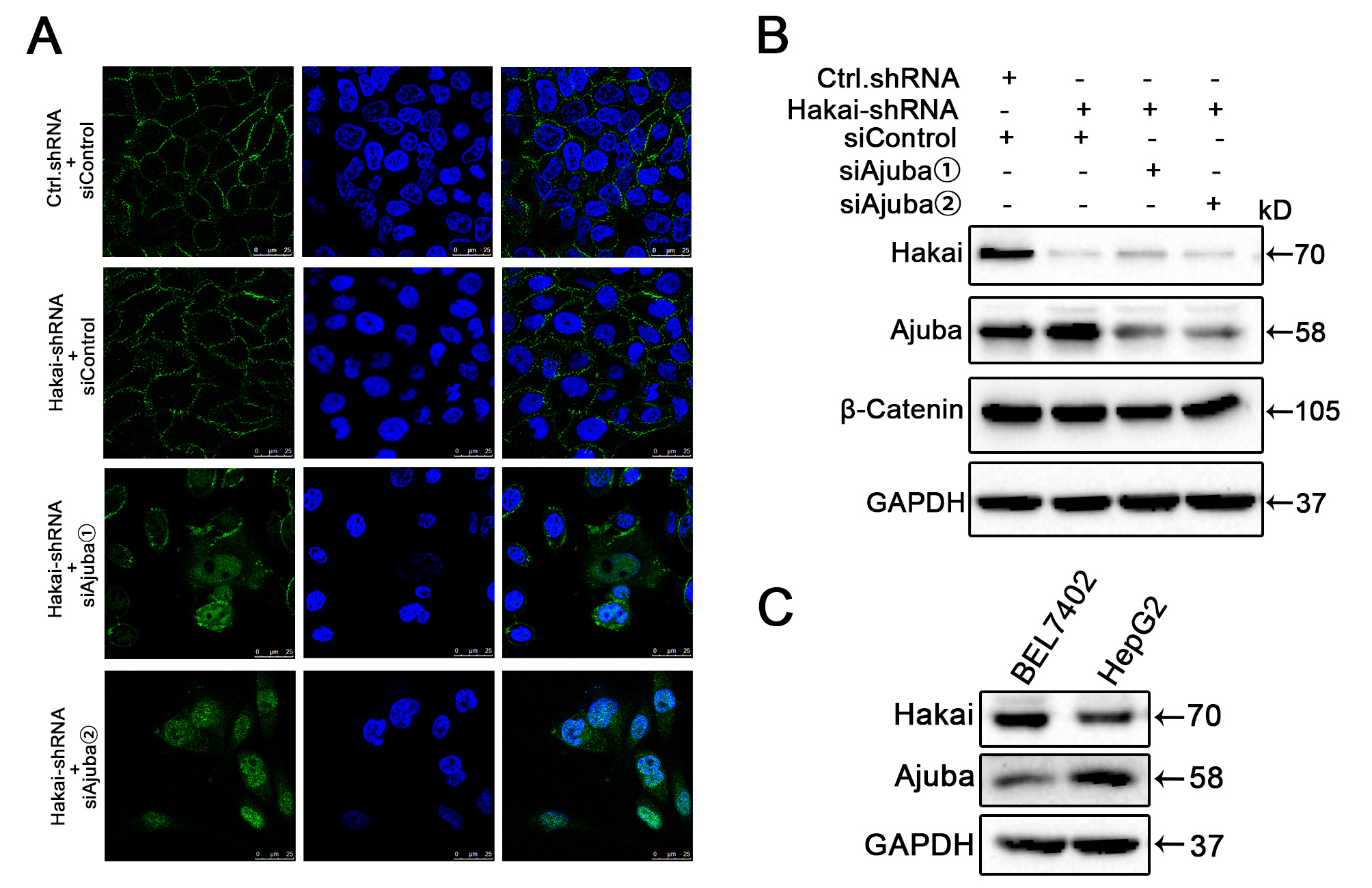

Supplement: Supplementary file 5 — Figure S5. Ajuba knockdown-mediated β-catenin translocation into nucleus is not dependent on Hakai. (A, B) HepG2 cells were transfected with specific siRNAs to silence Ajuba protein in Hakai-depleted HepG2 cells. β-catenin translocation were tested by confocal assay, Scale bar = 25 μm (A). The expression of Ajuba, Hakai and β-catenin were tested by immunoblot assay, GAPDH was used as a loading control (B). (C) Immunoblot analysis of Ajuba and Hakai in BEL7402 and HepG2 cell lysis. GAPDH was used as a loading control. (JPG 617 kb) [file 13046_2018_806_MOESM5_ESM.jpg]

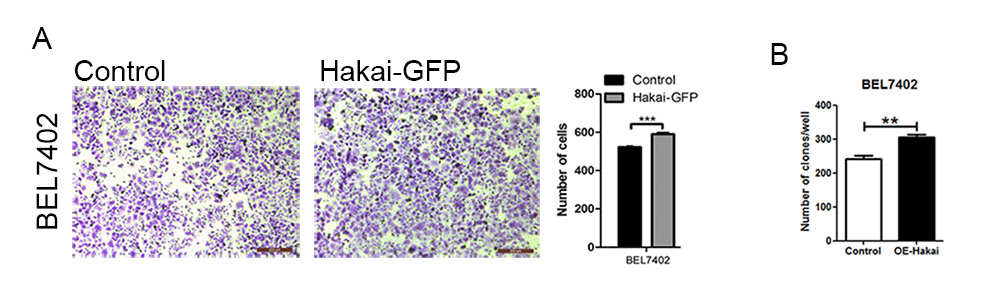

Supplement: Supplementary file 6 — Figure S6. Hakai promotes BEL7402 cells invasion and growth. (A) Representative images and quantification of invasion in GFP-tagged Hakai-overexpressing BEL7402 cells by adenovirus. Scale bar = 200 μm. (B) Analysis of the ability of Hakai-overexpressing BEL7402 cells by adenovirus to form colonies. Data are presented as Mean ± SEM from three independent experiments (**p < 0.01, ***p < 0.001). (JPG 146 kb) [file 13046_2018_806_MOESM6_ESM.jpg]
